# Association of pro-inflammatory diet with increased risk of gallstone disease: a cross-sectional study of NHANES January 2017–March 2020

**DOI:** 10.3389/fnut.2024.1344699

**Published:** 2024-03-14

**Authors:** Jinnian Cheng, Qian Zhuang, Weiyi Wang, Ji Li, Lu Zhou, Ying Xu, Haiqin Zhang, Zixu Zhang, Fengli Zhou, Daming Yang, Yimin Chu, Haixia Peng

**Affiliations:** ^1^Digestive Endoscopy Center, Tongren Hospital, Shanghai Jiaotong University School of Medicine, Shanghai, China; ^2^Digestive Endoscopy Center, Shanghai Sixth People's Hospital Affiliated to Shanghai Jiao Tong University School of Medicine, Shanghai, China

**Keywords:** National Health and Nutrition Examination Survey (NHANES), gallstone disease, pro-inflammatory diet, dietary inflammatory index (DII), population-based study

## Abstract

**Background and aim:**

Gallstone disease (GSD) is a major public health problem worldwide. The dietary inflammatory index (DII) and the energy-adjusted DII (E-DII) have been used to describe dietary inflammatory potential. The current study sought to investigate the pro-inflammatory role of diet on GSD among outpatients in the United States.

**Methods:**

Cross-sectional data from 7,334 individuals older than 20 years who participated in the National Health and Nutrition Examination Survey (NHANES) from January 2017 to March 2020 were obtained. The relationship between GSD and DII was assessed using self-reported data. An association between DII and the risk of GSD was determined using sample-weighted logistic regression and restricted cubic splines (RCS). Subgroup analyzes were conducted to assess the interaction between DII and related factors. Sensitivity analysis was further used to confirm the stability of the relationship. To control for the effect of total energy intake, E-DII was calculated and analyzed.

**Results:**

A total of 10.5% of the study participants had GSD. The DII ranged from −5.52 to 5.51, and the median DII was significantly higher for participants with GSD than those without (1.68 vs. 1.23, *p* < 0.001). There was a significant and stable positive relationship between DII and GSD in adjusted models (OR 1.10, 95% CI 1.00–1.20). In the fully adjusted model, subjects with DII scores in the highest tertile were more likely to have GSD than those in the lowest tertile (OR 1.52, 95% CI 1.19–1.93). An apparent dose–response association between DII and GSD was detected. The association between E-DII and GSD remained stable.

**Conclusion:**

Higher DII/E-DII scores linked to the intake of a pro-inflammatory diet were positively associated with a higher risk of GSD. These findings suggest that pro-inflammatory dietary patterns can promote the formation of gallstones.

## 1 Introduction

Gallstone disease (GSD) is common in the general population and its incidence has increased in recent years. The prevalence rate of GSD among adults in Europe, the United States, and other developed countries is approximately 10–15% (1). More than 20% of patients with GSD will develop symptoms, including colic or infection, during their lifetime (2). The direct and indirect costs of GSD are a leading cause of gastrointestinal disease-related hospitalization, resulting in a significant economic burden on families and society (3, 4).

Based on their composition, gallstones can be classified into cholesterol stones, which account for >90% of all gallstones, and other stones represented by black and brown pigments (2). Gallstone formation is shown to be multifactorial (1, 5) and risk factors include age, ethnicity, genetics, female gender, and lifestyle. GSD is also linked to insulin resistance, obesity, metabolic syndrome, and diabetes, of which diet plays a vital role. Studies indicate that a high intake of cholesterol, fatty acids, carbohydrates, and legumes can increase the risk of GSD. In contrast, the consumption of unsaturated fat, coffee, fiber, ascorbic acid (vitamin C), and calcium may lower the risk of this condition (5, 6). The specific dietary pattern that contributes to the development of GSD remains poorly understood, however.

It is well established that inflammation plays an essential role in the formation of gallstones (7–9). Inflammatory processes can affect cholesterol and bile acid metabolism by changing the metabolism of protein and fat, increasing the level of bile salt, and promoting the formation of gallstones (9). While GSD is linked to inflammation, evidence on whether a pro-inflammatory diet increases the risk of GSD remains limited.

Several nutritional indices such as Diet Inflammatory Index (DII), Dietary Antioxidant Index (DAI), Dietary Phytochemical Index (DPI), Nutrition Index (NI), dietary insulin index, Dietary Approaches to Stop Hypertension (DASH), and Mediterranean diet (MED) were reported to evaluate the effect of diet on chronic diseases (10–16). Among these evaluation indicators, the DII was originally proposed in 2009 and recalculated in 2014 to quantify the potential inflammatory level of individual dietary components by giving them a score ranging from maximum anti-inflammatory to maximum pro-inflammatory (17, 18). In addition, to adjust the influence of total energy intake, the energy-adjusted DII (E-DII) was developed (19). The DII/E-DII index has been verified by several inflammatory biomarkers, including C-reactive protein (CRP), tumor necrosis factor (TNF-a), and interleukin-I (IL). In the past decade, this index has been widely used to explore the relationship between anti- or pro-inflammatory diets and disease morbidity and mortality (13, 14, 16, 20–22). However, only a few studies have explored the specific relationship between an inflammatory diet and the development of GSD.

The current study sought to assess the cross-sectional relationship between DII/E-DII and GSD using data from the National Health and Nutrition and Examination Surveys (NHANES). We found that exposure to a pro-inflammatory diet would increase the risk of GSD.

## 2 Methods

### 2.1 Data sources

Data were obtained from individuals who participated in the National Health and Nutrition Examination Survey (NHANES) from January 2017 to March 2020. NHANES is a stratified multi-stage sampling survey conducted by the National Center for Health Statistics (NCHS) and designed to assess the health and nutrition status of Americans. The survey, which includes a family interview and a health examination, has been approved by the NCHS research ethics review board since 1999.

**Figure 1:**
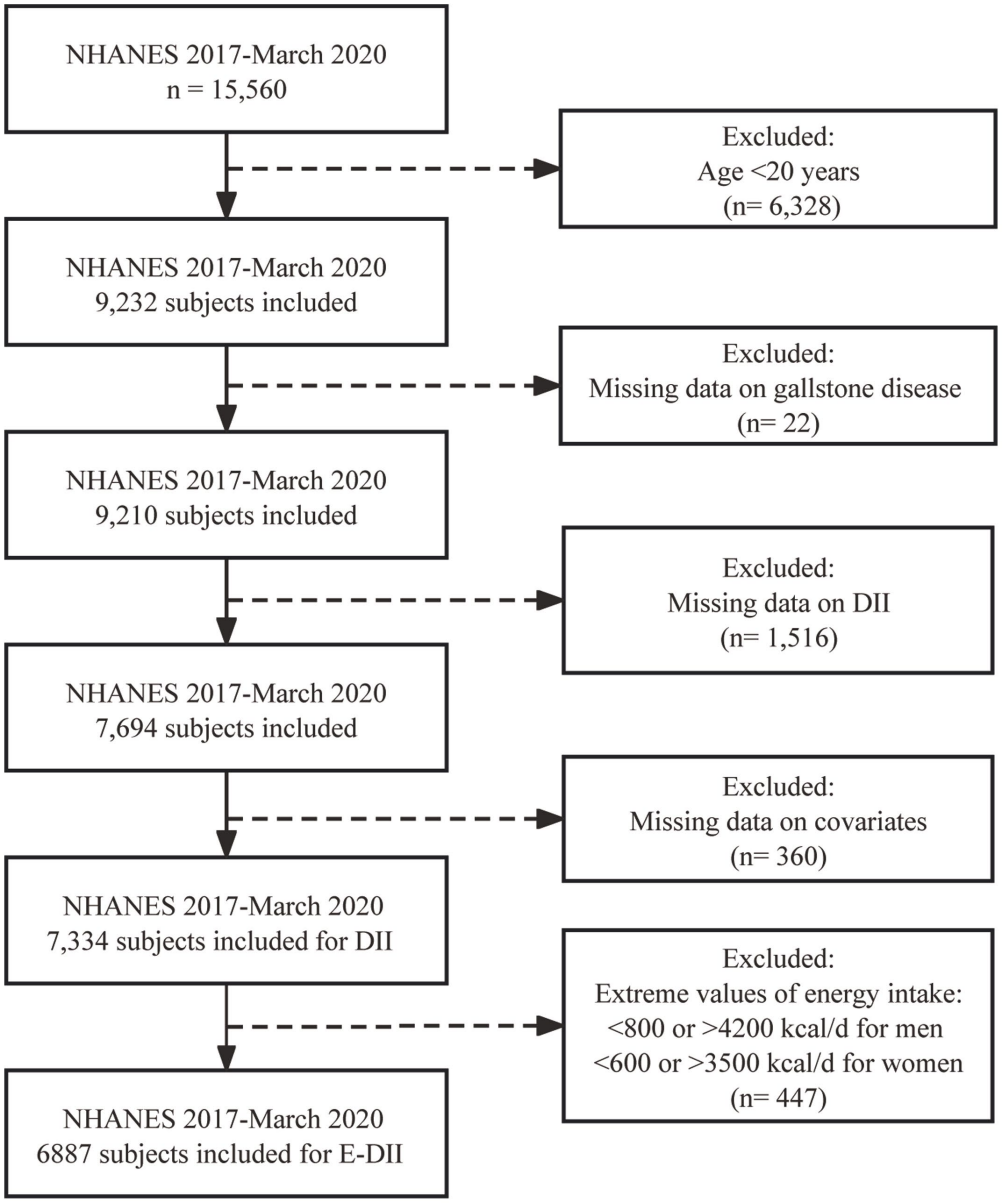
Flow diagram of study participant enrollment.

The current study included 15,560 individuals who participated in NHANES from January 2017–March 2020. After excluding individuals <20 years of age (*n* = 6,328), those missing data on GSD (*n* = 22), DII (*n* = 1,516), and covariates (*n* = 360), 7,334 participants were included in the final dataset for DII analysis. Extreme values of energy intake, including <800 kcal/d or > 4,200 kcal/d for men and < 600 kcal/d or > 3,500 kcal/d for women, were excluded when calculating the E-DII (n = 447) (23, 24). The inclusion and exclusion processes are shown in Figure 1.

### 2.2 Definition of GSD

The presence or absence of GSD is dependent on a patient’s self-report response to the question: “Has a doctor or other health professional ever told you that you had gallstones?”

### 2.3 Dietary inflammation index calculation

The DII is a potential tool to assess the anti- or pro-inflammatory quality of an individual’s diet by calculating the total potential inflammatory level of the dietary components consumed. This study calculated the exact nutritional intake of each participant using the nutritional intake information that was collected on day 1 and stored in the NHANES diet database. A total of 28 nutrients, including alcohol, vitamin A/B1/B2/B6/B12/C/D/E, β-carotene, caffeine, carbohydrate, cholesterol, energy, total fat, fiber, folic acid, iron, magnesium, zinc, selenium, monounsaturated fatty acids, niacin, n-3 fatty acids, n-6 fatty acids, protein, polyunsaturated fatty acids, and saturated fat, were used to determine the DII score in this study (Supplementary Table S2).

The specific calculation scheme of DII referred to the research of Shivappa et al. (18). Firstly, the dietary consumption information was compared to a worldwide daily intake database. The Z-score of each nutrient component was calculated based on the standard global daily mean intake and deviation (SD) values. Then it was transformed into a centered percentile, and multiplied by the respective overall inflammatory effect score to obtain the food parameter-specific DII score. Finally, all of the food parameter-specific DII scores were summated to gain an overall DII score for each individual. A higher DII score indicates that a diet is more pro-inflammatory in nature while a lower DII score indicates that a diet is more anti-inflammatory.

Accounting for the effect of total energy intake, density method was used to make energy adjustments for food and nutrient intake so that each parameter was expressed per thousand kilocalories (1,000 kcal). Then, the steps similar to the DII calculation were repeated to obtain E-DII but using an energy-adjusted global database (19).

### 2.4 Covariate assessment

Based on prior studies (1, 2, 25, 26), several potential confounding variables were selected as covariates for the analysis. The following demographic information was obtained: age (<40, 40–60, >60 years), gender (male or female), and race (non-Hispanic Black, non-Hispanic White, Mexican American, Other Hispanic, and Other). Body mass index (BMI) was calculated using height and weight data obtained during the NHANES mobile physical examination. Patients with a BMI >30 were categorized as obese while those ≤30 were categorized as non-obese. Participants were considered sedentary if they had ≥600 min of sedentary activity in a typical day and categorized as non-sedentary if they had <600 min/d of sedentary activity. Smoking and drinking status were classified according to the participants’ self-reported questionnaire responses. A respondent was defined as a non-smoker if they had smoked <100 cigarettes in their lifetime, and defined as a former smoker if they had smoked ≥100 cigarettes in their lifetime but did not smoke currently. Individuals who reported still smoking every day or on some days were defined as current smokers (27). Participants were further categorized as non-drinkers, light drinkers (1 to <30 drinks/month), or heavy drinkers (≥30 drinks/month). Diabetes, fatty liver, thyroid disease, and history of cancer were defined based on self-reported responses or confirmed clinical diagnoses (28–30).

### 2.5 Statistical analysis

Sample design and weights for the complex multi-stage cluster survey were considered using the Centers for Disease Control and Prevention (CDC) guidelines for the analysis of NHANES data. Participant characteristics were presented as means with standard deviation, SD for continuous variables, and the unweighted number of participants and weighted percentages (%) for categorical variables. Continuous variables were compared among groups using the Wilcoxon rank-sum test for complex survey samples and categorical variables were compared among groups using a weighted Chi-square test.

Sample-weighted logistic regression models were used to calculate odds ratios (ORs) and 95% confidence intervals (CIs) were used to measure associations between DII/E-DII scores and GSD. Four models were used after analyzing and adjusting for confounding factors. Model 1 represented the unadjusted crude model, model 2 was adjusted for sociodemographic variables (age group, sex, race, and ethnicity), and model 3 was based on model 2 and further adjusted for health-related and lifestyle factors, including sedentary activity, obesity, alcohol drinking status, smoking status, fatty liver, diabetes, and thyroid disease. To avoid over-adjustment, metabolic syndrome was defined as the presence of obesity, fatty liver, and/or diabetes (31). Model 4 was based on model 2 and further adjusted for sedentary activity, alcohol drinking status, smoking status, thyroid disease, and metabolic syndrome.

Restricted cubic splines were used to assess the dose–response relationship between GSD and DII scores, using four knots at prespecified locations according to the 5th, 35th, 65th, and 95th DII score percentiles. Subgroup analyzes were conducted using stratified multivariate regression analysis to assess the interaction between DII scores and specific covariates. *p* values for interactions across subgroups were calculated using the likelihood ratio test.

Given that the inclusion of the element of alcohol in the DII calculation, and data on alcohol consumption (*n* = 205) accounted for the largest proportion of the population participants with missing covariates (*n* = 360), sensitivity analyzes were performed to assess the robustness of the associations between DII and GSD after excluding alcohol intake (*n* = 7,539).

All statistical analyzes were performed with R software version 4.2.3 (R Core Team, Vienna, Austria. http://www.r-project.org/) using the survey package, version 4.1–1. All statistical tests were two-sided, and significance was considered at *p* < 0.05.

## 3 Results

### 3.1 Participant characteristics

A total of 7,334 participants were included in the study analyzes for DII. The general characteristics of the participants with and without GSD are shown in Table 1. Of these, 771 and 6,563 participants did and did not have GSD, respectively, for a prevalence of 10.5%. The DII scores ranged from −5.52 (highly anti-inflammatory) to +5.51 (highly pro-inflammatory). Participants with GSD had a significantly higher DII score than those without (1.68 vs. 1.23, *p* < 0.001, Table 2). Participants with GSD were older, more likely to be female, and had a higher BMI value than those without (all *p* < 0.001). Sedentary activity, alcohol drinking status, smoking status, fatty liver, diabetes, and thyroid disease were also significantly associated with GSD (all *p* < 0.05).

**Table 1:** Characteristics of January 2017–March 2020 NHANES participants.

Characteristic	Overall *n* = 7,334	Without GSD *n* = 6,563 (89.5%)	With GSD *n* = 771 (10.5%)	*p-*value
Age, years	48.3 (17.4)	47.3 (17.2)	57.6 (15.8)	<0.001
Sex				<0.001
Male	3,593 (48.5%)	3,368 (51.0%)	225 (27.5%)	
Female	3,741 (51.5%)	3,195 (49.0%)	546 (72.5%)	
Race/ethnicity				0.068
Non-Hispanic White	2,659 (62.8%)	2,320 (62.2%)	339 (67.9%)	
Non-Hispanic Black	1,949 (11.3%)	1,793 (11.8%)	156 (7.3%)	
Other	1,137 (9.9%)	1,047 (10.0%)	90 (9.0%)	
Mexican American	849 (8.3%)	754 (8.4%)	95 (7.6%)	
Other Hispanic	740 (7.7%)	649 (7.6%)	91 (8.1%)	
Drinking status				0.003
Non/Light drinker	6,304 (83.2%)	5,603 (82.3%)	701 (90.4%)	
Heavy drinker	1,030 (16.8%)	960 (17.7%)	70 (9.6%)	
Smoking status				0.004
Non-smoker	4,223 (57.5%)	3,814 (58.1%)	409 (52.1%)	
Former smoker	1,775 (25.5%)	1,535 (24.7%)	240 (32.5%)	
Current smoker	1,336 (17.0%)	1,214 (17.2%)	122 (15.5%)	
Sedentary activity, min/day	350.0 (199.6)	347.1 (197.5)	375.2 (215.7)	0.011
BMI, kg/m^2^	29.8 (7.2)	29.4 (6.9)	33.3 (8.6)	<0.001
Fatty liver				<0.001
No	4,609 (63.7%)	4,215 (64.9%)	394 (53.8%)	
Yes	2,725 (36.3%)	2,348 (35.1%)	377 (46.2%)	
Diabetes				<0.001
No	5,885 (85.3%)	5,362 (86.5%)	523 (74.9%)	
Yes	1,449 (14.7%)	1,201 (13.5%)	248 (25.1%)	
Thyroid disease				<0.001
No	6,470 (87.9%)	5,865 (89.3%)	605 (76.3%)	
Yes	864 (12.1%)	698 (10.7%)	166 (23.7%)	
Metabolic syndrome				<0.001
No	4,027 (58.4%)	3,711 (59.8%)	316 (46.2%)	
Yes	3,307 (41.6%)	2,852 (40.2%)	455 (53.8%)	

BMI, body mass index; GSD, gallstone disease.

Data are presented as unweighted numbers (weighted percentages) for categorical variables and means (SDs) for continuous variables.

**Table 2:** Dietary inflammatory index (DII) scores for January 2017–March 2020 NHANES participants with and without gallstone disease.

Characteristic	Overall *n* = 7,334	Without GSD *n* = 6,563(89.5%)	With GSD *n* = 771(10.5%)	*p-*value
DII	1.29 (−0.43, 2.66)	1.23 (−0.46, 2.63)	1.68 (0.01, 2.91)	<0.001
DII group				<0.001
Tertile 1	1,111 (17.2%)	1,022 (17.9%)	89 (11.2%)	
Tertile 2	3,977 (54.5%)	3,591 (54.6%)	386 (54.0%)	
Tertile 3	2,246 (28.3%)	1,950 (27.5%)	296 (34.9%)	

DII, dietary inflammatory index; GSD, gallstone disease.

In further, the E-DII scores ranged from −5.25 to +5.33. There was no statistically significant difference of E-DII score between the two groups in the univariate analysis (Supplementary Table S1).

### 3.2 Association between DII/E-DII score and GSD risk

**Figure 2:**
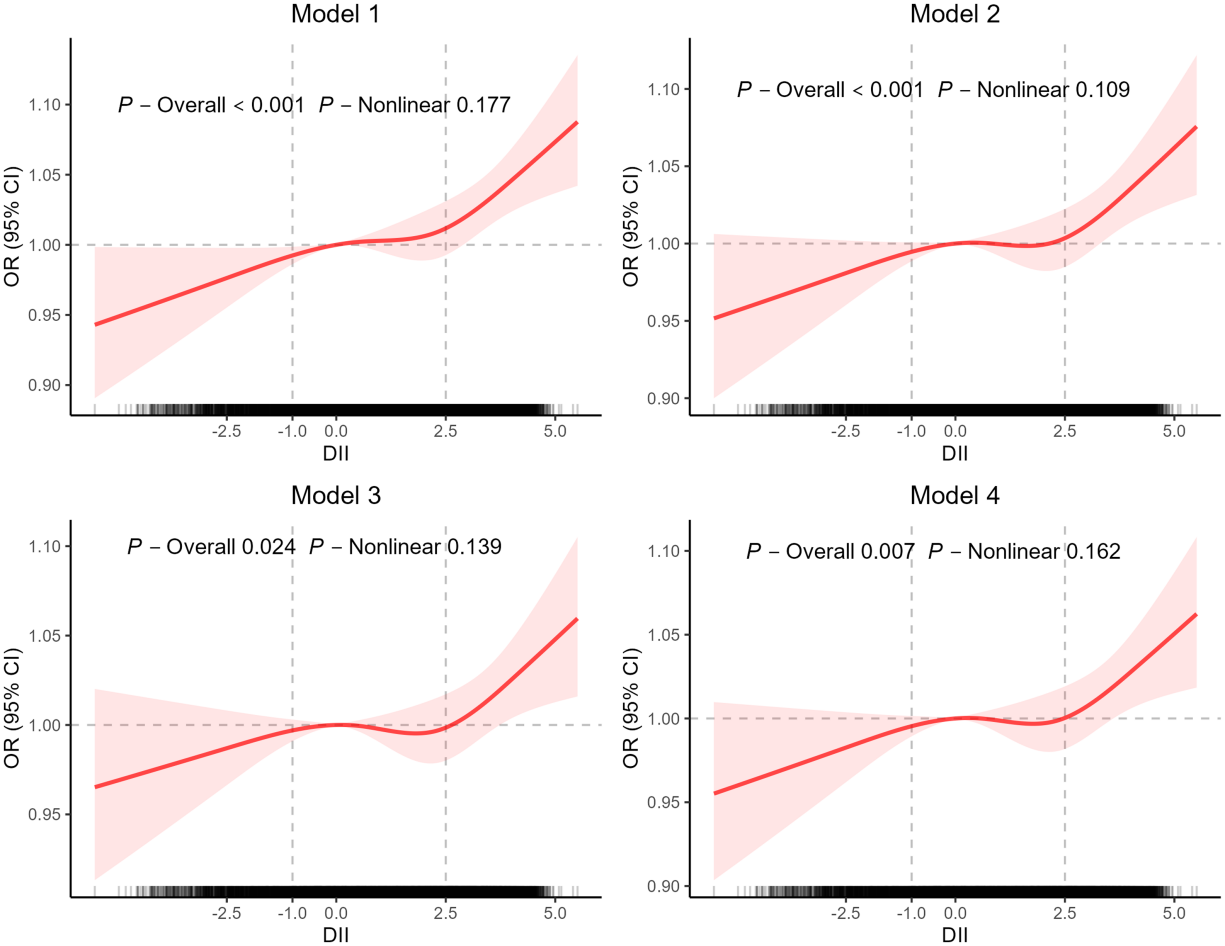
The restricted cubic spline for the association between dietary inflammatory index (DII) scores and gallstone disease (GSD).

The association between the DII/E-DII score and the risk of GSD was determined using a sample-weighted multivariable logistic regression model (Table 3) and remained stable in each adjusted model. A higher DII score was associated with an increased risk of GSD (model 1, OR 1.22, 95% CI 1.12–1.32; model 2, OR 1.16, 95% CI 1.06–1.27; model 3, OR 1.10, 95% CI 1.00–1.20; model 4, OR 1.12, 95% CI 1.02–1.22). After full adjustment (model III), DII was associated with the presence of gallstones (OR 1.10, 95% CI 1.00–1.20). This association remained statistically significant after DII scores were grouped into tertiles. Subjects with the highest tertile DII scores had a higher risk of GSD than those with the lowest tertile DII scores (OR 1.52, 95% CI 1.19–1.93). The data also indicated that there was a linear relationship between DII scores and GSD (*p* for trend <0.05). Furthermore, multivariable-adjusted restricted cubic spline regression demonstrated a significant dose–response relationship between DII scores and the risk of GSD (Figure 2).

**Table 3:** Association between DII/E-DII and the presence of gallstone disease (GSD) among January 2017–March 2020 NHANES participants.

Characteristic	Model 1		Model 2		Model 3		Model 4	
	OR	95% CI	OR	95% CI	OR	95% CI	OR	95% CI
*sqrt-trans DII, per 1 SD*	1.22	1.12, 1.32	1.16	1.06, 1.27	1.10	1.00, 1.20	1.12	1.02, 1.22
*Categorical DII*								
Tertile 1	Ref.		Ref.		Ref.		Ref.	
Tertile 2	1.58	1.15, 2.17	1.42	1.03, 1.94	1.34	0.95, 1.90	1.39	1.00, 1.93
Tertile 3	2.03	1.54, 2.68	1.74	1.37, 2.21	1.52	1.19, 1.93	1.59	1.25, 2.02
*p* for trend		<0.001		<0.001		0.004		0.002
*sqrt-trans E-DII, per 1 SD*	1.02	0.92, 1.14	1.15	1.03, 1.29	1.08	0.96, 1.22	1.11	0.98, 1.25
*Categorical E-DII*								
Tertile 1	Ref.		Ref.		Ref.		Ref.	
Tertile 2	1.45	1.03, 2.03	1.71	1.23, 2.37	1.55	1.08, 2.22	1.65	1.17, 2.33
Tertile 3	1.61	1.07, 2.42	2.33	1.61, 3.36	1.90	1.26, 2.84	2.07	1.36, 3.16
*p* for trend		0.024		<0.001		0.007		0.003

OR, Odds Ratio; CI, Confidence Interval; DII, dietary inflammatory index; E-DII, energy-adjusted DII.

Sample-weighted logistic regression models were used.

Model 1: unadjusted.

Model 2: adjusted for sociodemographic variables (age group, sex, race and ethnicity).

Model 3: fully adjusted model: Model 2+ sedentary activity, obesity, alcohol drinking status, smoking status, fatty liver, diabetes, and thyroid disease.

Model 4: Model 2+ sedentary activity, alcohol drinking status, smoking status, thyroid disease and metabolic syndrome.

Similar results of E-DII with GSD were obtained when grouped into tertiles. Individuals with the highest tertile E-DII scores had a higher risk of GSD than those with the lowest tertile E-DII scores (model 1, OR 1.61, 95% CI 1.07–2.42; model 2, OR 2.33, 95% CI 1.61–3.36; model 3, OR 1.90, 95% CI 1.26–2.84; model 4, OR 2.07, 95% CI 1.36–3.16) (Table 3).

### 3.3 Subgroup analyzes

**Figure 3:**
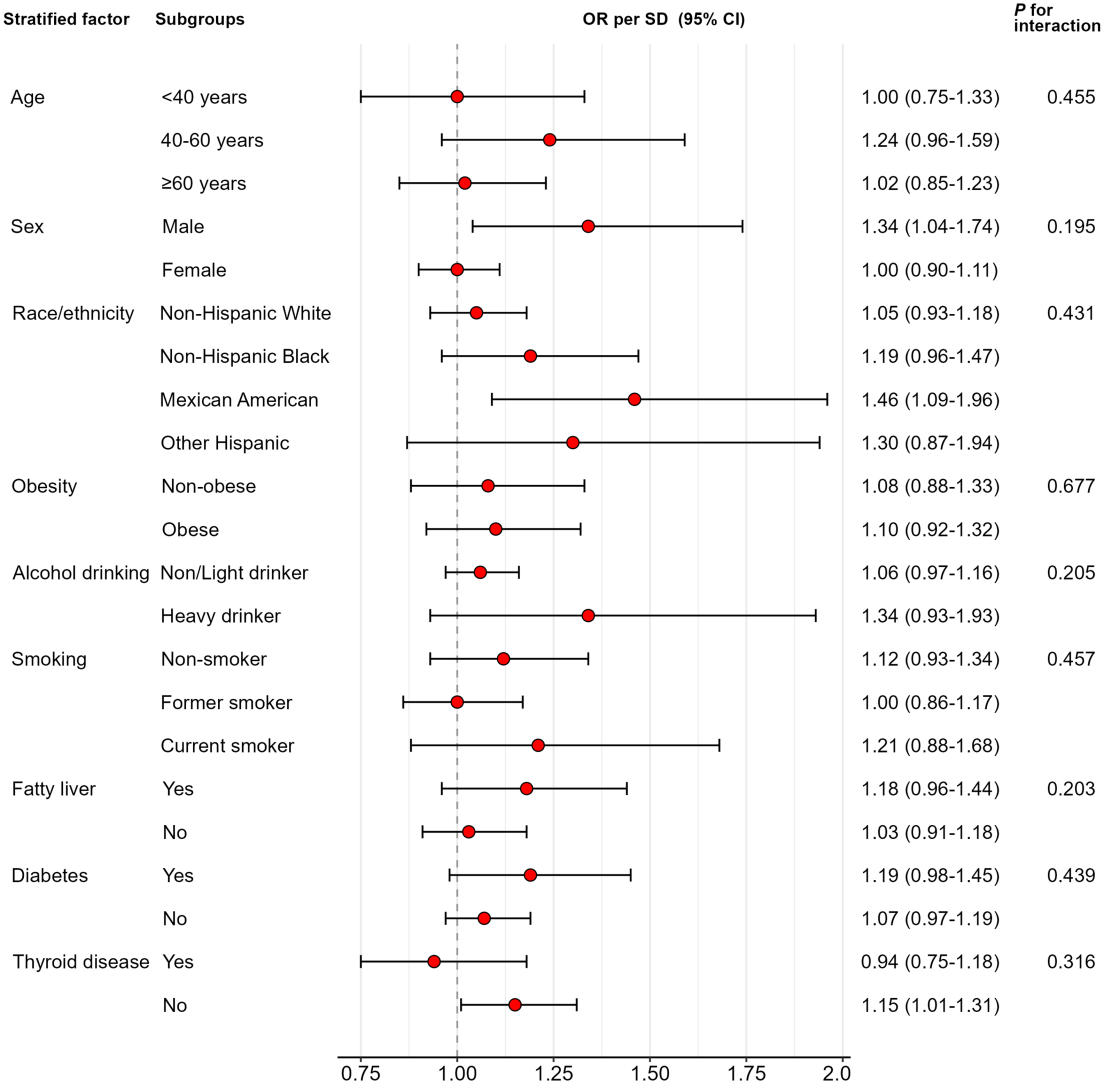
Subgroup analyzes of the association between dietary inflammatory index (DII) and the development of gallstone disease (GSD). OR, odds ratio; CI, confidence interval. Sample-weighted logistic regression models were applied. Each stratification was adjusted for confounding factors such as age group, sex, race and ethnicity, sedentary activity, obesity, alcohol drinking status, smoking status, fatty liver, diabetes, and thyroid disease except the stratification factor itself.

Results of the subgroup analyzes are shown in Figure 3. No significant interactions were identified (*p* for interaction >0.1 for all). Effect of DII on GSD was consistent across all nine pre-specified subgroups.

### 3.4 Sensitivity analyzes

Excluding alcohol intake did not reduce the statistical significance of the relationship between DII score and GSD in any of the models (model 1, OR 1.22, 95% CI 1.12–1.32; model 2, OR 1.16, 95% CI 1.06–1.27; model 3, OR 1.11, 95% CI 1.01–1.23; model 4 OR 1.14, 95% CI 1.03–1.26) (Table 4).

**Table 4:** Association between dietary inflammatory index (DII) and the presence of gallstone disease (GSD) among January 2017–March 2020 NHANES participants, excluding alcohol intake.

Characteristic	Model 1		Model 2		Model 3		Model 4	
	OR	95% CI	OR	95% CI	OR	95% CI	OR	95% CI
*sqrt-trans DII, per 1 SD*	1.22	1.12, 1.32	1.16	1.06, 1.27	1.11	1.01, 1.23	1.14	1.03, 1.26
*Categorical DII*								
Tertile 1	Ref.		Ref.		Ref.		Ref.	
Tertile 2	1.49	1.09, 2.05	1.35	0.97, 1.87	1.29	0.91, 1.83	1.35	0.97, 1.88
Tertile 3	1.94	1.47, 2.55	1.67	1.30, 2.15	1.51	1.16, 1.96	1.60	1.23, 2.09
*p* for trend		<0.001		<0.001		0.006		0.002

OR, Odds Ratio; CI, Confidence Interval.; DII, dietary inflammatory index.

Sample-weighted logistic regression models were used.

Model 1: unadjusted.

Model 2: adjusted for sociodemographic variables (age group, sex, race, and ethnicity).

Model 3: fully adjusted model: Model 2+ sedentary activity, obesity, smoking status, fatty liver, diabetes, and thyroid disease.

Model 4: Model 2+ sedentary activity, smoking status, thyroid disease, and metabolic syndrome.

## 4 Discussion

This study investigated the association between DII scores and GSD using data from a nationally representative study, NHANES. A robust association between DII score and GSD was observed in US adults, indicating that a pro-inflammatory diet is positively associated with an increased risk of GSD. After adjusting for all confounding factors, individuals with the highest DII/E-DII scores were shown to be at higher risk of developing GSD than those with the lowest DII/E-DII scores (OR 1.52, 1.19–1.93 95% CI, p trend <0.05 for DII, OR 1.90, 95% CI 1.26–2.84 for E-DII, Table 3). A dose–response relationship was observed between DII scores and GSD risk using restricted cubic spline regression. This association was generally consistent across subgroups. Sensitivity analysis confirmed the robustness of the primary analysis.

In recent years, the role of diet in regulating inflammation and affecting health has received widespread attention. The DII, developed by Shivappa et al. (18), is a reliable quantitative tool for evaluating the effects of diet on health by linking inflammatory cytokine levels in the blood to the outcomes of various chronic diseases (19). It was based on six of the most commonly studied inflammatory markers including IL-1β, IL-4, IL-6, IL-10, CRP, and TNF-α, and is used to quantitatively evaluate the anti- and pro-inflammatory effects of food (18, 19). A pro-inflammatory diet, that is, the higher DII score, is associated with an increased risk of several chronic noncommunicable diseases (NCD) (14, 20, 32–34), including metabolic syndrome and related diseases, cardiovascular and cerebrovascular diseases, cancers of various anatomic sites and depression and other mental health outcomes.

To the best of our knowledge, this is the first large population-based cross-sectional study to explore the association between a pro-inflammatory diet and GSD risk among a US population. One previous cross-sectional study, conducted using the Dena cohort, examined the association between DII scores and GSD (35). In contrast to findings from the current study, this report found that a pro-inflammatory diet was associated with a reduced risk of GSD. Due to the population restrictions of the geographic area, most of the participants had a similar diet and DII scores, which ranged from −0.4 to 1.43, so highly pro- or anti-inflammatory dietary data were lacking. Other studies that have synthesized and reviewed global database information have found that when the DII index covers all 45 food parameters, scores could range from −8.87 to +7.98. When it only refers to 25–30 parameters, the theoretical range of DII is usually from −5.5 to +5.5 (18, 19). Thus, the prior cross-sectional study may not be representative of people who consume a wide range of rich diets. While DII scores in the current study ranged from −5.52 (most anti-inflammatory) to +5.51 (most pro-inflammatory), which was consistent with most previous findings (18, 19). In addition, compared to the 4.3% incidence of GSD in the prior cross-sectional study (median DII −0.08), the GSD incidence in the current study was 10.5% (median DII 1.29). This reflects a likely correlation between the consumption of a pro-inflammatory diet and the development of GSD. Another case–control study was consistent with our results, in which the higher DII score, serum inflammatory and oxidative stress biomarkers were related to higher risk of GD in Iranian women (36). In our research, the E-DII was further calculated and analyzed for adjusting the effect of total energy intake, which indicated a stable and consistent correlation between E-DII and GSD.

Cholelithiasis is a critical public health issue and current researches suggest that three major pathogenic abnormalities are involved in the formation of gallstones: supersaturated gallbladder bile, precipitation and nucleation of excess cholesterol, and gallbladder hypomotility (1). Previous studies indicated that inflammation played an important role in the formation of gallstones (9, 37, 38). Higher levels of circulating inflammatory proteins and cytokines, including IL-1α, IL-6, IL-8, IL-10, IL-12 (p70), IL-13, CRP and tumor necrosis factor (TNF-α), were significantly associated with the increased risk of GSD (9, 37, 39–41). Inflammation-related histopathological changes occur in the gallbladder wall prior to the formation of cholesterol gallstones in both animal models and humans (37, 38, 42).Pro-inflammatory diet may increase the levels of circulating inflammatory proteins and cytokines in serum, which contributes to gallbladder wall fibrosis, and the impairment of gallbladder contractility (42). In addition, pro-inflammatory cytokines may lead to mucin hypersecretion, which plays an important role in the cholesterol nucleation process (41). The gallbladder hypomotility and mucin-related cholesterol nucleation predispose to the formation of gallstones (37, 43). The biological mechanisms underlying the association between pro-inflammatory diet and GSD would benefit from further researches.

This study had still several limitations. Firstly, given the cross-sectional study design of NHANES, the causal relationship between DII/E-DII scores and GSD could not be determined. Secondly, dietary data, GSD, and confounding factors were obtained from interviews or patient self-report questionnaires in the NHANES database, and are associated with an inevitable recall bias. Finally, while a sensitivity analysis was conducted, several participants were excluded due to the lack of data, which may have impacted the findings. A well-designed prospective cohort study will be necessary to explore the deeper relationship between DII/E-DII scores and GSD.

## 5 Conclusion

In conclusion, our findings indicate that a pro-inflammatory diet, that is, higher DII/E-DII scores, was positively associated with a higher risk of GSD. These findings indicate that pro-inflammatory dietary patterns can promote the formation of gallstones. Active dietary management and intervention should be considered to prevent the development of GSD.

## Data availability statement

The datasets presented in this study can be found in online repositories. The names of the repository/repositories and accession number(s) can be found at: https://www.cdc.gov/nchs/nhanes/about_nhanes.htm.

## Ethics statement

The studies involving humans were approved by the National Center for Health Statistics Institutional Review Board and Ethics Review Board. The studies were conducted in accordance with the local legislation and institutional requirements. The participants provided their written informed consent to participate in this study.

## Author contributions

JC: Writing – original draft, Conceptualization, Methodology, Funding acquisition. QZ: Software, Writing – original draft, Methodology. WW: Writing – review & editing, Conceptualization, Data curation. JL: Formal analysis, Resources, Writing – review & editing. LZ: Methodology, Visualization, Writing – review & editing. YX: Data curation, Writing – review & editing. HZ: Resources, Writing – review & editing. ZZ: Formal analysis, Visualization, Writing – review & editing. FZ: Investigation, Validation, Writing – review & editing. DY: Investigation, Validation, Writing – review & editing. YC: Project administration, Writing – review & editing, Funding acquisition. HP: Project administration, Writing – review & editing, Funding acquisition.

## References

[1] Di Ciaula A Wang DQ Portincasa P. An update on the pathogenesis of cholesterol gallstone disease. Curr Opin Gastroenterol. (2018) 34:71–80. doi: 10.1097/MOG.0000000000000423, PMID: 29283909 PMC8118137

[2] Lammert F Gurusamy K Ko CW Miquel JF Mendez-Sanchez N Portincasa P. Gallstones. Nat Rev Dis Primers. (2016) 2:16024. doi: 10.1038/nrdp.2016.24 27121416

[3] Everhart JE Ruhl CE. Burden of digestive diseases in the United States part iii: liver, biliary tract, and pancreas. Gastroenterology. (2009) 136:1134–44. doi: 10.1053/j.gastro.2009.02.038, PMID: 19245868

[4] Everhart JE Ruhl CE. Burden of digestive diseases in the United States part I: overall and upper gastrointestinal diseases. Gastroenterology. (2009) 136:376–86. doi: 10.1053/j.gastro.2008.12.015, PMID: 19124023

[5] Stinton LM Shaffer EA. Epidemiology of gallbladder disease: cholelithiasis and cancer. Gut Liver. (2012) 6:172–87. doi: 10.5009/gnl.2012.6.2.172, PMID: 22570746 PMC3343155

[6] Davidović DB Tomić DV Jorg JB. Dietary habits as a risk factor of gallstone disease in Serbia. Acta Chir Iugosl. (2011) 58:41–4. doi: 10.2298/ACI1104041D, PMID: 22519190

[7] Fremont-Rahl JJ Ge Z Umana C Whary MT Taylor NS Muthupalani S. An analysis of the role of the indigenous microbiota in cholesterol gallstone pathogenesis. PLoS One. (2013) 8:e70657. doi: 10.1371/journal.pone.0070657, PMID: 23923015 PMC3726617

[8] Shabanzadeh DM Skaaby T Sørensen LT Eugen-Olsen J Jørgensen T. Metabolic biomarkers and gallstone disease - a population-based study. Scand J Gastroenterol. (2017) 52:1270–7. doi: 10.1080/00365521.2017.1365166, PMID: 28799434

[9] Liu Z Kemp TJ Gao YT Corbel A McGee EE Wang B. The Association of Circulating Inflammation Proteins and Gallstone Disease. J Gastroenterol Hepatol. (2018) 33:1920–4. doi: 10.1111/jgh.14265, PMID: 29671891 PMC7576672

[10] Arabshahi V Amiri R Ghalishourani SS Hasaniani N Nozarian S Tavasolian R. Association between dietary insulin index and load with cardiometabolic risk factors and risk of metabolic syndrome among the patients with type 2 diabetes: a cross-sectional study. BMC Nutr. (2023) 9:141. doi: 10.1186/s40795-023-00803-z, PMID: 38049837 PMC10694962

[11] Rahimlou M Grau N Banaie-Jahromi N Taheri M Khosravi A Mavrommatis Y. Association of Adherence to the dietary approach to stop hypertension and Mediterranean diets with blood pressure in a non-hypertensive population: results from Isfahan salt study (Iss). Nutr Metab Cardiovasc Dis. (2022) 32:109–16. doi: 10.1016/j.numecd.2021.09.029 34893410

[12] Kim M Park K. Association between phytochemical index and metabolic syndrome. Nutr Res Pract. (2020) 14:252–61. doi: 10.4162/nrp.2020.14.3.252, PMID: 32528632 PMC7263893

[13] Petermann-Rocha F Wirth MD Boonpor J Parra-Soto S Zhou Z Mathers JC. Associations between an inflammatory diet index and severe non-alcoholic fatty liver disease: a prospective study of 171,544 Uk biobank participants. BMC Med. (2023) 21:123. doi: 10.1186/s12916-023-02793-y, PMID: 37013578 PMC10071692

[14] Ruiz-Canela M Bes-Rastrollo M Martínez-González MA. The role of dietary inflammatory index in cardiovascular disease, metabolic syndrome and mortality. Int J Mol Sci. (2016) 17:1265. doi: 10.3390/ijms17081265, PMID: 27527152 PMC5000663

[15] Xu Q Qian X Sun F Liu H Dou Z Zhang J. Independent and joint associations of dietary antioxidant intake with risk of post-stroke depression and all-cause mortality. J Affect Disord. (2023) 322:84–90. doi: 10.1016/j.jad.2022.11.013, PMID: 36372128

[16] Jayanama K Theou O Godin J Cahill L Shivappa N Hébert JR. Relationship between diet quality scores and the risk of frailty and mortality in adults across a wide age Spectrum. BMC Med. (2021) 19:64. doi: 10.1186/s12916-021-01918-5, PMID: 33722232 PMC7962372

[17] Cavicchia PP Steck SE Hurley TG Hussey JR Ma Y Ockene IS. A new dietary inflammatory index predicts interval changes in serum high-sensitivity C-reactive protein. J Nutr. (2009) 139:2365–72. doi: 10.3945/jn.109.114025, PMID: 19864399 PMC2777480

[18] Shivappa N Steck SE Hurley TG Hussey JR Hébert JR. Designing and developing a literature-derived, population-based dietary inflammatory index. Public Health Nutr. (2014) 17:1689–96. doi: 10.1017/s1368980013002115, PMID: 23941862 PMC3925198

[19] Hébert JR Shivappa N Wirth MD Hussey JR Hurley TG. Perspective: the dietary inflammatory index (dii)-lessons learned, improvements made, and future directions. Adv Nutr. (2019) 10:185–95. doi: 10.1093/advances/nmy071, PMID: 30615051 PMC6416047

[20] Wang X Sun M Wang L Li J Xie Z Guo R. The role of dietary inflammatory index and physical activity in depressive symptoms: results from Nhanes 2007-2016. J Affect Disord. (2023) 335:332–9. doi: 10.1016/j.jad.2023.05.012, PMID: 37172657

[21] Sun M Fang J Gao W He Y Ma Y Jin L. Association of the Dietary Inflammatory Index with phenotypic age. Epidemiol Health. (2023) 45:e2023051. doi: 10.4178/epih.e2023051, PMID: 37170498 PMC10593589

[22] Farazi M Jayedi A Shab-Bidar S. Dietary inflammatory index and the risk of non-communicable chronic disease and mortality: an umbrella review of meta-analyses of observational studies. Crit Rev Food Sci Nutr. (2023) 63:57–66. doi: 10.1080/10408398.2021.1943646, PMID: 34176394

[23] Chen L Ming J Chen T Hébert JR Sun P Zhang L. Association between dietary inflammatory index score and muscle mass and strength in older adults: a Study from National Health and nutrition examination survey (Nhanes) 1999-2002. Eur J Nutr. (2022) 61:4077–89. doi: 10.1007/s00394-022-02941-9, PMID: 35809101 PMC9596556

[24] Shi Y Lin F Li Y Wang Y Chen X Meng F. Association of pro-Inflammatory Diet with increased risk of all-cause dementia and Alzheimer's dementia: a prospective study of 166,377 Uk biobank participants. BMC Med. (2023) 21:266. doi: 10.1186/s12916-023-02940-5, PMID: 37480061 PMC10362711

[25] Cai JS Qiang S Bao-Bing Y. Advances of recurrent risk factors and Management of Choledocholithiasis. Scand J Gastroenterol. (2017) 52:34–43. doi: 10.1080/00365521.2016.1224382, PMID: 27610642

[26] Konyn P Alshuwaykh O Dennis BB Cholankeril G Ahmed A Kim D. Gallstone disease and its association with nonalcoholic fatty liver disease, all-cause and cause-specific mortality. Clin Gastroenterol Hepatol. (2023) 21:940–8.e2. doi: 10.1016/j.cgh.2022.04.043, PMID: 35643414

[27] Sutton JD Salas Martinez ML Gerkovich MM. Environmental tobacco smoke and periodontitis in United States non-smokers, 2009 to 2012. J Periodontol. (2017) 88:565–74. doi: 10.1902/jop.2017.160725, PMID: 28168902

[28] Association AD. Diagnosis and classification of diabetes mellitus. Diabetes Care. (2010) 33:S62–9. doi: 10.2337/dc10-S062, PMID: 20042775 PMC2797383

[29] Siddiqui MS Vuppalanchi R Van Natta ML Hallinan E Kowdley KV Abdelmalek M. Vibration-controlled transient elastography to assess fibrosis and steatosis in patients with nonalcoholic fatty liver disease. Clin Gastroenterol Hepatol. (2019) 17:156–63.e2. doi: 10.1016/j.cgh.2018.04.043, PMID: 29705261 PMC6203668

[30] Eddowes PJ Sasso M Allison M Tsochatzis E Anstee QM Sheridan D. Accuracy of Fibroscan controlled attenuation parameter and liver stiffness measurement in assessing steatosis and fibrosis in patients with nonalcoholic fatty liver disease. Gastroenterology. (2019) 156:1717–30. doi: 10.1053/j.gastro.2019.01.042, PMID: 30689971

[31] Kassi E Pervanidou P Kaltsas G Chrousos G. Metabolic syndrome: definitions and controversies. BMC Med. (2011) 9:48. doi: 10.1186/1741-7015-9-48, PMID: 21542944 PMC3115896

[32] Hariharan R Odjidja EN Scott D Shivappa N Hébert JR Hodge A. The dietary inflammatory index, obesity, type 2 diabetes, and cardiovascular risk factors and diseases. Obes Rev. (2022) 23:e13349. doi: 10.1111/obr.13349, PMID: 34708499

[33] Fowler ME Akinyemiju TF. Meta-analysis of the association between dietary inflammatory index (dii) and cancer outcomes. Int J Cancer. (2017) 141:2215–27. doi: 10.1002/ijc.30922, PMID: 28795402 PMC6056732

[34] Fan L Zhao S Shi H Zhang S. Role of Bmi in the relationship between dietary inflammatory index and non-alcoholic fatty liver disease: an intermediary analysis. Scand J Gastroenterol. (2023) 58:1159–65. doi: 10.1080/00365521.2023.2213791, PMID: 37211749

[35] Sadri Z Harouni J Vahid F Khosravani Z Najafi F. Association between the dietary inflammatory index with gallstone disease: finding from Dena Persian cohort. BMJ Open Gastroenterol. (2022) 9:e000944. doi: 10.1136/bmjgast-2022-000944, PMID: 36123004 PMC9486214

[36] Liu N Feng Y Li J Ma X Ma F. Relationship between the dietary inflammatory index and kidney stone prevalence. World J Urol. (2022) 40:1545–52. doi: 10.1007/s00345-022-03998-1, PMID: 35396944

[37] Maurer KJ Carey MC Fox JG. Roles of infection, inflammation, and the immune system in cholesterol gallstone formation. Gastroenterology. (2009) 136:425–40. doi: 10.1053/j.gastro.2008.12.031, PMID: 19109959 PMC2774219

[38] Rege RV. Inflammatory cytokines Alter human gallbladder epithelial cell absorption/secretion. J Gastrointest Surg. (2000) 4:185–92. doi: 10.1016/s1091-255x(00)80055-4, PMID: 10675242

[39] Ghorbani M Hekmatdoost A Darabi Z Sadeghi A Yari Z. Dietary inflammatory index and risk of gallstone disease in Iranian women: a case-control study. BMC Gastroenterol. (2023) 23:311. doi: 10.1186/s12876-023-02943-9, PMID: 37710148 PMC10500896

[40] Liu T Siyin ST Yao N Duan N Xu G Li W. Relationship between high-sensitivity C reactive protein and the risk of gallstone disease: results from the Kailuan cohort study. BMJ Open. (2020) 10:e035880. doi: 10.1136/bmjopen-2019-035880, PMID: 32963062 PMC7509952

[41] Maurer KJ Rao VP Ge Z Rogers AB Oura TJ Carey MC. T-cell function is critical for murine cholesterol gallstone formation. Gastroenterology. (2007) 133:1304–15. doi: 10.1053/j.gastro.2007.07.005, PMID: 17919501 PMC2043485

[42] van Erpecum KJ Wang DQ Moschetta A Ferri D Svelto M Portincasa P. Gallbladder histopathology during murine gallstone formation: relation to motility and concentrating function. J Lipid Res. (2006) 47:32–41. doi: 10.1194/jlr.M500180-JLR200, PMID: 16224116

[43] Reynoso-Paz S Coppel RL Mackay IR Bass NM Ansari AA Gershwin ME. The immunobiology of bile and biliary epithelium. Hepatology. (1999) 30:351–7. doi: 10.1002/hep.510300218, PMID: 10421640

