# Supplementary material for: Association of pro-inflammatory diet with increased risk of gallstone disease: a cross-sectional study of NHANES January 2017–March 2020
Source: Front Nutr. 2024 Mar 14;11:1344699. doi: 10.3389/fnut.2024.1344699 (PMC10972905; doi:10.3389/fnut.2024.1344699)
Supplement: Supplementary file 1 [file Table_1.DOCX]

**Supplementary Table 1. E-DII scores for January 2017–March 2020 NHANES participants with and without gallstone disease.**

| **Characteristic** | **Overall**  n = 6887 | **Without GSD**  n = 6150(89.3%) | **With GSD**  n = 737(10.7%) | ***P-*value** |
| --- | --- | --- | --- | --- |
| DII | 1.16 (-0.34, 2.35) | 1.17 (-0.33, 2.34) | 1.07 (-0.41, 2.46) | >0.9 |
| DII group |  |  |  | 0.044 |
| Tertile 1 | 787 (13.1%) | 716 (13.5%) | 71 (9.5%) |  |
| Tertile 2 | 4,373 (63.1%) | 3,903 (63.0%) | 470 (64.0%) |  |
| Tertile 3 | 1,727 (23.8%) | 1,531 (23.5%) | 196 (26.5%) |  |
| Abbreviations: E-DII, energy-adjusted dietary inflammatory index; GSD, gallstone disease. | | | | |
