# Supplementary material for: Association of pro-inflammatory diet with increased risk of gallstone disease: a cross-sectional study of NHANES January 2017–March 2020
Source: Front Nutr. 2024 Mar 14;11:1344699. doi: 10.3389/fnut.2024.1344699 (PMC10972905; doi:10.3389/fnut.2024.1344699)
Supplement: Supplementary file 2 [file Table_2.DOCX]

**Supplementary Table 2. Characteristics of nutrients for calculating dietary inflammatory index (DII)**

| **Characteristic** | **Overall**  n = 7334(100.0%) | **Tertile 1**  n = 1111(17.2%) | **Tertile 2**  n = 3977(54.5%) | **Tertile 3**  n = 2246(28.3%) | **P value** |
| --- | --- | --- | --- | --- | --- |
| Alcohol (g) | 11 (27) | 14 (28) | 12 (29) | 6 (20) | <0.001 |
| Vitamin B12 (μg) | 4.8 (5.7) | 7.3 (8.6) | 5.0 (5.2) | 2.8 (3.2) | <0.001 |
| Vitamin B6 (mg) | 2.15 (2.19) | 3.40 (2.33) | 2.27 (2.26) | 1.15 (1.36) | <0.001 |
| β-Carotene (μg) | 2,437 (4,485) | 6,750 (7,554) | 1,999 (3,271) | 656 (984) | <0.001 |
| Caffeine (mg) | 170 (216) | 179 (210) | 173 (212) | 159 (227) | 0.006 |
| Carbohydrate (g) | 245 (124) | 335 (155) | 251 (109) | 178 (89) | <0.001 |
| Cholesterol (mg) | 315 (255) | 409 (330) | 333 (242) | 224 (191) | <0.001 |
| Energy (kcal) | 2,150 (983) | 2,962 (1,239) | 2,241 (803) | 1,480 (612) | <0.001 |
| Total fat (g) | 88 (49) | 123 (65) | 93 (41) | 59 (30) | <0.001 |
| Fibre (g) | 17 (10) | 30 (12) | 17 (8) | 9 (4) | <0.001 |
| Folic acid (μg) | 160 (170) | 245 (250) | 163 (161) | 101 (78) | <0.001 |
| Fe (mg) | 14 (8) | 22 (11) | 14 (7) | 8 (4) | <0.001 |
| Mg (mg) | 304 (150) | 499 (166) | 311 (99) | 173 (62) | <0.001 |
| MUFA (g) | 30 (18) | 43 (24) | 32 (15) | 20 (10) | <0.001 |
| Niacin (mg) | 26 (18) | 37 (20) | 28 (17) | 16 (11) | <0.001 |
| Protein (g) | 81 (42) | 116 (50) | 86 (35) | 52 (24) | <0.001 |
| PUFA (g) | 21 (14) | 31 (19) | 22 (12) | 12 (7) | <0.001 |
| Riboflavin (mg) | 2.08 (1.33) | 3.06 (1.51) | 2.16 (1.25) | 1.33 (0.84) | <0.001 |
| Saturated fat (g) | 29 (17) | 37 (24) | 30 (16) | 21 (13) | <0.001 |
| Se (μg) | 113 (63) | 155 (81) | 120 (56) | 74 (36) | <0.001 |
| Thiamin (mg) | 1.58 (0.95) | 2.38 (1.23) | 1.64 (0.81) | 0.99 (0.48) | <0.001 |
| Vitamin A (RE) | 632 (626) | 1,318 (940) | 585 (457) | 305 (216) | <0.001 |
| Vitamin C (mg) | 77 (88) | 163 (121) | 73 (74) | 32 (41) | <0.001 |
| Vitamin D (μg) | 4.3 (5.4) | 7.8 (8.7) | 4.4 (4.7) | 2.2 (2.2) | <0.001 |
| Vitamin E (mg) | 9.4 (6.7) | 17.1 (9.1) | 9.5 (4.8) | 4.6 (2.4) | <0.001 |
| Zn (mg) | 10.8 (6.9) | 16.1 (9.0) | 11.2 (6.2) | 6.7 (3.7) | <0.001 |
| n-3 Fatty acids (g) | 2.06 (1.66) | 3.33 (2.46) | 2.16 (1.35) | 1.09 (0.79) | <0.001 |
| n-6 Fatty acids (g) | 19 (12) | 28 (17) | 20 (11) | 11 (7) | <0.001 |
| Means (SDs) for continuous variables.  Wilcoxon rank-sum test for complex survey samples was used. | | | | | |
